# Prolificacy Assessment of Spermatozoan via State-of-the-Art Deep Learning Frameworks

**DOI:** 10.1109/access.2022.3146334

**Published:** 2022-01-26

**Authors:** SATISH CHANDRA, MAHENDRA KUMAR GOURISARIA, HARSHVARDHAN GM, DEBANJAN KONAR, XIN GAO, TIANYANG WANG, MIN XU

**Affiliations:** 1 School of Computer Engineering, KIIT Deemed to be University, Bhubaneswar, Odisha 751024, India; 2 CASUS—Center for Advanced Systems Understanding, Helmholtz-Zentrum Dresden-Rossendorf (HZDR), 02826 Görlitz, Germany; 3 Computer, Electrical and Mathematical Science and Engineering Division, King Abdullah University of Science and Technology, Thuwal 23955, Saudi Arabia; 4 Department of Computer Science & Information Technology, Austin Peay State University, Clarksville, TN 37044, USA; 5 Computational Biology Department, School of Computer Science, Carnegie Mellon University, Pittsburgh, PA 15213, USA

**Keywords:** Sperm abnormality, deep learning, transfer learning

## Abstract

Childlessness or infertility among couples has become a global health concern. Due to the rise in infertility, couples are looking for medical supports to attain reproduction. This paper deals with diagnosing infertility among men and the major factor in diagnosing infertility among men is the Sperm Morphology Analysis (SMA). In this manuscript, we explore establishing deep learning frameworks to automate the classification problem in the fertilization of sperm cells. We investigate the performance of multiple state-of-the-art deep neural networks on the MHSMA dataset. The experimental results demonstrate that the deep learning-based framework outperforms human experts on sperm classification in terms of accuracy, throughput and reliability. We further analyse the sperm cell data by visualizing the feature activations of the deep learning models, providing a new perspective to understand the data. Finally, a comprehensive analysis is also demonstrated on the experimental results obtained and attributing them to pertinent reasons.

## I. INTRODUCTION

The deprivation from pregnancy after a year of carefully timed and unprotected intercourse is referred to as infertility. Recently, it has been witnessed that approximately 15 percent of couples worldwide are suffering from infertility problems. The most common factor of involuntary childlessness is the male factor of infertility, which is the cause for almost 30 to 40 percent of infertile couples [1], [2]. The problem may be related to poor sperm motility (asthenospermia), low sperm production (oligospermia), or abnormal morphology (teratozoospermia) [3]. One or more combinations of these problems are used for classifying the different forms of male factor infertility [4]. A spermatozoa morphology abnormality is one of the problems for male factor infertility, which shows oligoasthenoteratozoospermia [5].

However, it is also a daunting task to find an evident cause for the male factor infertility for a large number of andrological disorders. Hence, logical treatments are still lacking and unchecked approaches are still being prescribed to the infertile man just on an empirical basis which is often questionable [6]. Medical or surgical treatments are also given to patients with varicocele, infections, obstructions, hypogonadism and cryptorchidism [7].

Infertility diagnosis and treatment have a major challenge of performing consistent and rapid analysis of sperm images [8]. The male factor infertility is guided by evaluating the spermatic plasma features and sperm specifications such as sperm morphology and pH, concentration, viscosity and motility [9], [10]. Sperm morphology is very critical for detecting abnormal sperms and anomaly types. According to the World Health Organization (WHO), abnormalities related to sperm heads are tapered, amorphous, round, small, large, pyriform, small acrosome, large acrosome, vacuolated and two-headed [10], [11]. These abnormalities are present in different shapes, sizes and textures, making the classification difficult [12]. The sperm morphology determination has been improved by the Automated Sperm Morphometric Analysis (ASMA) with higher accuracy [9].

The widely accepted Intra-Cytoplasmic Sperm Injection (ICSI) technology [13] among the medical expert communities demands accurate and quick classification of sperm. The procedure for selecting the sperm for the intra-cytoplasmic morphologically picked sperm injection (IMSI) is executed at very high magnification (6000x). However, common laboratories have relatively low magnification power (400x and 600x) [14]. In addition, the clinical assessment is very time-consuming and based on the perception of the embryologist which may be inexact, non-repeatable, subjective and hard to teach. Hence, the Computer-Assisted Semen Analysis (CASA) has carried forward a breakthrough in analyzing and selecting the best semen for the ICSI procedure with a higher level of standardization and automation [15]. This technology has improved the fertilization and pregnancy rates [9], [16]. In this research work, we have studied various deep learning architectures to automate the problem of classification in the fertilization of sperm cells.

The significant threefold contributions of our research work are provided below.

1. We present various deep learning architectures for noticing malformation present in human sperm morphology. Deep convolutional neural networks are trained to classify the sperm cell into positive (normal) and negative (abnormal) for different morphological features, *i.e.* vacuole, acrosome and head.
2. We also demonstrate deep learning and computer vision algorithms for visualizing and extracting sperm morphological features.
3. The main challenge lies in the fact that fewer images are available for the training and noisy images are taken using low magnification microscopes and imbalance class. To overcome this, a data augmentation technique has been applied to the dataset and deep neural network models with pre-trained weights have been employed to analyze the normality of the sperm vacuole, head and acrosome. Different neural networks have been visualized by neural activation and class activation maps.

The remainder section of the manuscript is arranged as follows. Section II discusses a compact literature review pertaining to sperm classification. The methodology and materials which include data exploration, data pre-processing, are presented in Section III. A brief introduction to various deep neural networks prevalent in sperm classification is discussed in Section III–B. The experimental setting of the deep learning models is detailed in Section IV. Rigorous experimental results and discussions are presented in Section V. Finally, conclusive remarks and future directions of research are confabulated in Section VI.

## II. RELATED WORK

With an advancement of the intra-cytoplasmic sperm injection (ICSI) technique, the research on sperm classification is an emerging topic in medical science. The classical machine learning algorithms have achieved notable importance in semen classification; however, it relies on manually crafted features and are then fed to the classifier [8]. Jiaqian *et al*. [17] employed the Principal Component Analysis (PCA) and Scale-invariant Feature Transform (SIFT) for the extraction of features (color, texture, shape and spatial features of the image) from the sperm images followed by the k-nearest neighbor (KNN) classifier and back-propagation neural network (BPNN). Shaker *et al*. [12] extracted pre-proposed features of the sperm (eg. length, area, perimeter, mass, etc.) from Olympus microscopic images with an x10 eyepiece and x100 objective and also proposed a new set of feature extractors, namely elliptical features for extracting the head contour. They used both the features with Linear Discriminant Analysis (LDA) classifier which significantly improved the distinction among the classes: Normal, Tapered, Pyriform and Amorphous.

A plethora of machine learning algorithms for sperm morphology analysis has been proposed in recent times. A notable example includes a two-staged-based structure for segmentation and detection of the nucleus and acrosome of human sperm cells by Chang *et al*. [18]. In the first stage, the sperm head is detected using k-means algorithm followed by mathematical morphology for candidate head refining. The sperm head, nucleus and acrosome are segmented accurately using histogram statistical analysis and clustering technique. They combined various color spaces, rather than using RBG color space and achieved an accuracy of 98% in detecting sperm heads. Shaker *et al*. [19] presented an adaptive patch-based dictionary learning (ADPL), where square smears are removed from semen. Class-specific dictionaries are learned from columnized smears from each sperm cell class. The best matching class is determined by the reconstruction of test image smears from class-specific dictionaries and evaluating them. They attained a sensitivity of 62% on the SCIAN-MorphoSpermGS dataset and a mean accuracy of 92.2% on the HuSHeM dataset.

Of late, Riordon *et al*. [8] demonstrated a deep neural network algorithm for classifying the sperm into the WHO categories (i.e, pyriform, tapered, normal and amorphous) for Scientific Image Analysis Gold-standard for Morphological Semen Analysis (SCIAN) dataset consisting of 1132 gray-scale images and Human Sperm Head Morphology (HuSHeM) dataset with 216 RGB images. They have reported an accuracy of 94.1% and outperforms the cascaded ensemble of support vector machines (CE-SVM) and Adaptive Patch-based Dictionary Learning (APDL) technique. Bijar *et al*. [9] proposed a fully automated recognition and discrimination technique to extract the nucleus, acrosome, tail and mid-piece from stained semen smear. A high magnification microscope (1000x) is employed for capturing the stained sperm cells images. Bayesian classifier along with Markov Random Field (MRF) and entropybased Expectation–Maximization (EM) algorithm [20], [21] are used for segmenting different parts of sperm cells and upgrading the apriori probability and Class Conditional Probability Density Function (CCPDF) of each class. The Structural Similarity Index (SSIM) is also incorporated for finding the pixels placed on the sperm’s tail. An automatic and faster algorithm relying on a deep learning network is suggested by Ghasemian *et al*. [22] to detect malformation using sperm cell images and gain an accuracy of 90%. This technique performs well in the present scenario with a minute magnification microscope (400x and 600x) and it can be pre-owned in the ICSI process. However, the proposed algorithm suffers from the extraction of the shape and size of the acrosome. Later on, Javadi *et al*. [5] proposed a deep learning algorithm for malformation recognition in male factor infertility abnormalities in sperm vacuole, acrosome and head on a collection of 1540 sperm cell images (MHSMA dataset [5]) and achieved an F-0.5 score of 83.86%, 84.74% and 94.65% in head, vacuole and acrosome malformation diagnosis, respectively.

## III. METHODOLOGY AND MATERIALS

**FIGURE 1.**
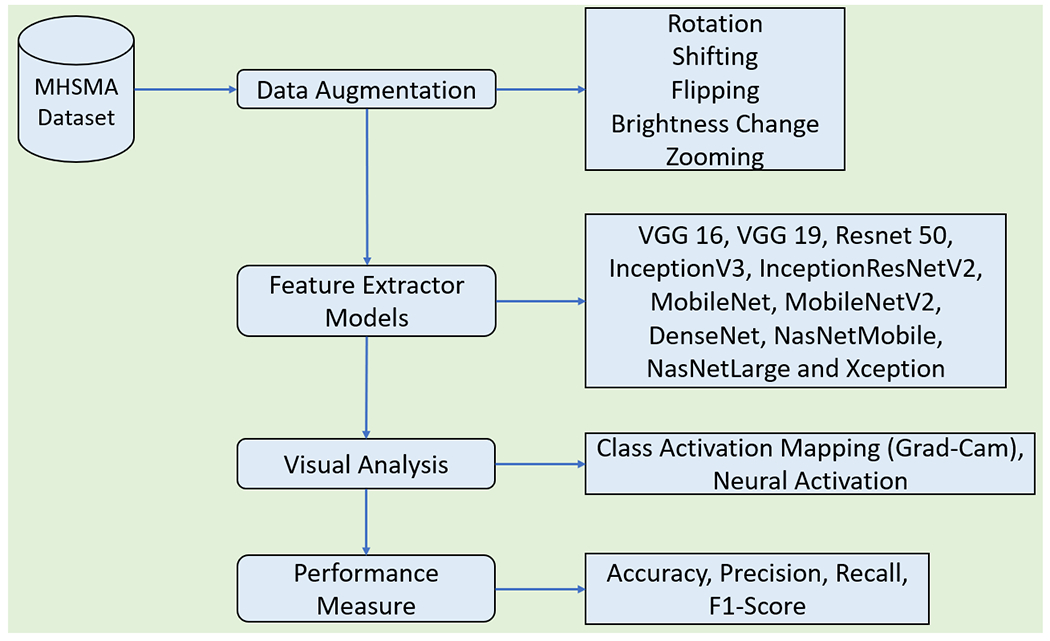
Workflow of sperm morphology analysis.

This research paper presents deep learning architectures for noticing malformation present in human sperm morphology. Deep convolutional neural networks are being trained for classifying the sperm cell into positive (normal) and negative (abnormal) for different morphological features *i.e.*, vacuole, acrosome and head. Figure 1 demonstrates the sperm morphology analysis algorithm as adopted in this work.

### A. DATA EXPLORATION

**FIGURE 2.**
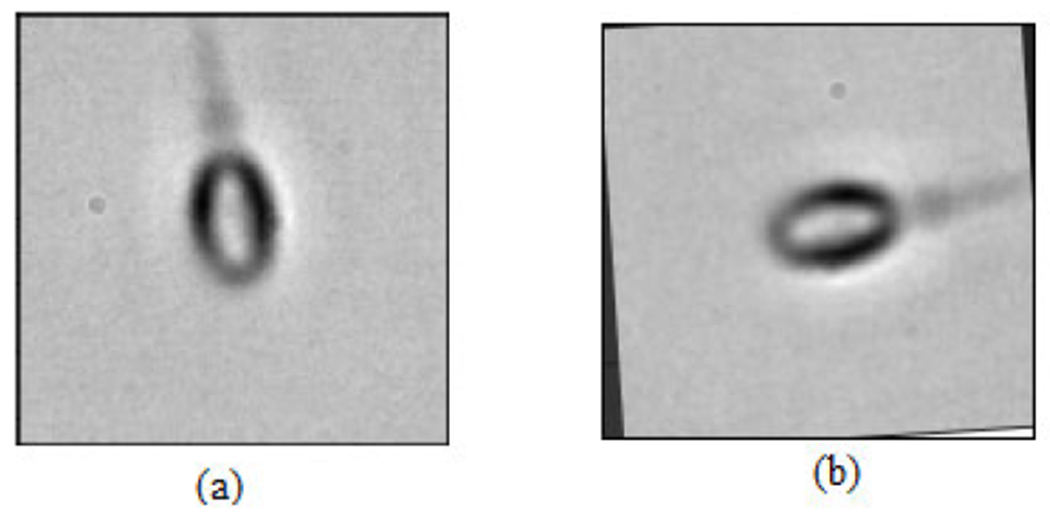
Data augmentation: (a) Original image (b) Rotated by 90 degree.

**FIGURE 3.**
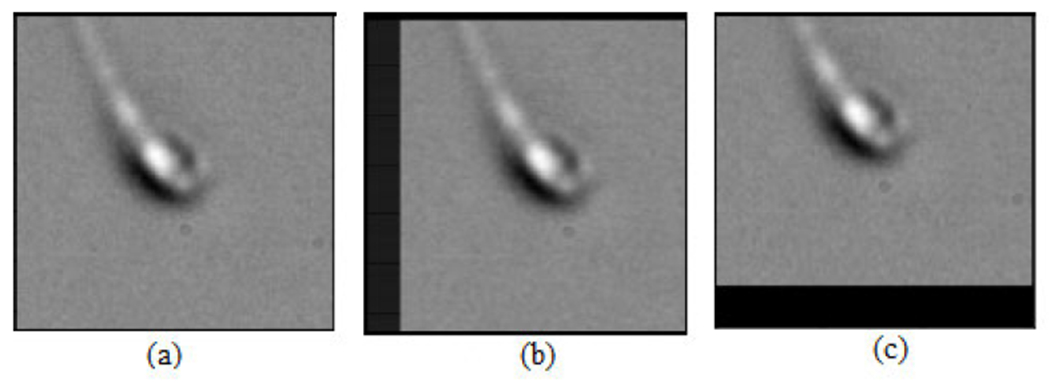
Data augmentation: (a) Original image (b) Horizontal shift and (c) Vertical shift.

**FIGURE 4.**
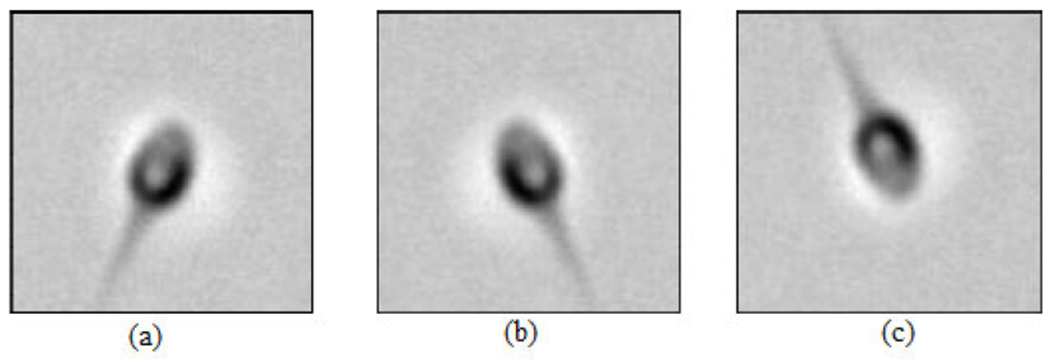
Data augmentation: (a) Original image (b) Horizontal flip and (c) Vertical flip.

In this work, the MHSMA [5] containing 1540 RGB images with a dimension of 1024 × 1280 pixels [22] has been used in the experiments. The MHSMA dataset [5] comprises 1540 gray-scale semen images with a dimension of 128 × 128 pixels and was acquired using an IX70 Olympus microscope which was equipped with a DP71 Olympus camera with the lens at a magnification of either 600× (834 images) or 400× (706 images). Each image is centered on the sperm’s head while capturing it. However, all the sample images in the dataset are overlapped among acrosome, vacuole and head. The deep learning models are trained, validated and tested using randomly chosen 1000, 240 and 300 images, respectively. The dataset [5] is split into training, validation and testing data with positive and negative samples. The complete illustration of the dataset has been provided in Table 1 and the dataset split ratios in training, validation and testing in Table 2. Due to the insufficiency of the training data, the trinity of data augmentation techniques (rotation, shifting and flipping) have been employed to virtually expand the dataset and intercept overfitting [23]. The rotation operation rotates the sperm cell image arbitrarily in the range of 0 to 90 as shown in Fig. 2. Horizontal shifting and vertical shifting have been performed in the range of 0.2 as shown in Fig. 3. The sperm cell images are flipped vertically (reflection) and horizontally (mirror) with a chance of 0.5 as shown in Fig. 4.

### B. DEEP LEARNING MODELS

In this study, the widely-used deep learning models, which have attained promising classification accuracy on the ImageNet dataset, are employed. Moreover, the pre-trained layers of the deep learning models are successfully implemented in this work with faster convergence. We have employed pre-trained deep learning models which include VGG16 [24], VGG19, ResNet50, InceptionV3 [23], InceptionResNetV2 [25], MobileNet [26], MobileNetV2 [27], DenseNet [28], NASNetMobile [29], NASNetLarge [30] and Xception [31] as a feature extractor. Theses deep learning networks are discussed in briefs in the following subsections.

#### 1) VGG16

VGG16 (Visual Geometry Group) is a deep convolutional neural network (DCNN) architecture presented by A. Zisserman and K. Simonyan [24]. This architecture has 3 fully connected layers and 13 convolutional layers. All the convolutional layers in VGG-16 are 3 × 3 layers with padding and stride size of 1 and the pooling layers are 2 × 2 layers with a stride size of 2. The feature map is diminished by 50 percent, after each pooling layer. The last pooling layer has 7 × 7 with 512 channels [32], [33]. The network comprises total of 14, 714, 688 parameters.

#### 2) VGG19

K. Simonyan and A. Zisserman also proposed the architecture of VGG19 in the year 2014 [24]. This architecture comprises 143 million parameters. VGG19 has 3 fully connected layers and 16 convolutional layers. It also has a max-pooling layer and a dropout layer. The model has a fixed input size of 224 × 224. The kernels are of size 3 × 3 with a stride of 1 and max-pooling is 2 × 2 layers with a stride of 2 [34].

#### 3) ResNet50

ResNet50 is a Deep Convolutional Neural Network (DCNN) model with 50 layers and it is based on Residual Network (ResNets) [35]. The elementary block is the bottleneck block having two rules: (i) the layers have equal units of the filter; and (ii) the units of filter are doubled if the feature map is reduced by 50%. The model is comprised of 5 stages and each stage has an identity block with 3 convolution layers and a convolution block also with 3 convolution layers. Downsampling has been performed by a convolutional layer with a stride of 2. A projection shortcut is used to equalize the size through a 1 × 1 convolution layer. The network concludes with thousand fully connected layers [36]. This architecture has 23 million parameters.

#### 4) INCEPTION-V3

Inception-V3 is a deep convolutional neural network model which is pre-trained on ImageNet datasets containing 1000 classes [23]. It is rethinking for early architecture after Inception-V1 and Inception-V2. It has a top-5 error rate of 3.5%. The model has a fixed input shape of size 299 × 299 RGB Image. It is 48 layers deep architecture with 23 million parameters [37].

#### 5) INCEPTION-V2

InceptionResNet-V2 [25] is a combination of two networks *i.e.*, residual networks [36] and Inception architecture [23]. The residual network is well known for its very deep architecture while the Inception model is popular for its numerous branch architectures. It comprises 164 layers of deep architecture that can classify images into 1000 categories. It has a fixed input shape of a size 299 × 299 RGB images. Each Inception block is followed by a filter expansion layer *i.e.*, 1 × 1 convolution layer used for scaling. On top of the traditional layer, batch-normalization is used [38].

#### 6) MobileNet

MobileNet [26] structure is built on depth-wise separable convolutions that factorize usual convolution into 1 × 1 point-wise convolution and depth wise convolution. Depth-wise convolution administers a sole filter to every input medium. A standard convolution combines and filters processes into a novel set of products in a sole step. The depth-wise separable convolution breaks this into duo layers, a distinct layer for combining and a distinct layer for filtering. All the layers of MobileNet are followed by RELU non-linearity and a batch norm except for the fully connected layer. It comprises a fixed input size of 224 × 224 RGB images. Counting point-wise convolutions and depth-wise as distinct layers, the MobileNet [26] structure have 28 layers with approximately 4 million parameters and top-5 accuracy of 89.5%.

#### 7) MobileNetV2

The fundamental structure of the MobileNetV2 [27] model is bottleneck depth-separable convolution with residuals. It holds the primary fully convolution layer with 32 filters, accompanied by 19 residual bottleneck layers. There are two distinct blocks each with 3 layers, a residual block of stride 1 and a block with stride 2 for downsizing. The initial layer is a 1 × 1 convolution layer with *RELU* followed by depth-wise convolution and a 1 × 1 convolution layer without nonlinearity. Kernel of size 3×3 has been used and also employed batch normalization and dropout. The internal output would have 384 (64×6) channels for the input with 64 channels [27].

#### 8) DenseNet

Huang *et al*. [28] proposed the DenseNet [28] architecture that is progressively hierarchical. In the DenseNet [28] structure, each layer directly connected to interior layers. It has a fixed input size of 224 × 224 RGB images. The DenseNet [28] constitutes the condensed network which provides an easy way to train efficient models. DenseNet [28] comprises multiple dense blocks containing multiple layers. A sequence of consecutive transformations has been performed by each layer. The initial transformation is a combination of rectified linear units and batch normalization (BN-ReLU). The growth rate explains how the dense model attains state-of-the-art outcomes. Bottleneck layers are initiated by employing a 1 × 1 convolution layer before 3 × 3 convolutions. Transition layers provide checks on the feature maps in a certain depth of the network, thereby improving the compactness of the entire network [39].

#### 9) NASNetMobile

NASNetMobile [29] is an expandable convolutional neural network architecture consisting of elementary building blocks which are improved by utilizing reinforcement learning [29]. A block consists of various distinguishable convolutions and pooling operations and is frequent many times according to the necessary volume of the network. It has strong feature extraction capabilities due to the repeated stacking of Inception and ResNet networks. It comprises 12 blocks with 564 million multiply-accumulates (MACs) and 5.3 million parameters. NASNetMobile [29] has various parameters that feature MobileNet, however, it outperforms in terms of accuracy. NASNet architecture consists of 2 different cells, *i.e.*, reduction cells that reduce the width and length of the input feature map by 50% of the original convolution layer and normal cells that do not change the dimension of the convolution layer [30].

#### 10) NASNetLarge

NasNetLarge [30] is a convolutional neural network architecture and it has a fixed input dimension of 331 × 331 RBG image. NASNetLarge [30] is a bigger architecture than NASNetMobile [29] and it has 88 million parameters. The model attained a top-5 accuracy of 96% on the ImageNet dataset.

#### 11) XCEPTION

Xception [31] is an extension of the Inception model and CNN architecture, fully relying on depth-wise separable convolution layers [31]. It may be noted that the mapping of the spatial correlations and cross-channel correlations in the feature map in convolutional neural networks can be completely decoupled. The model has 71 convolution layers and is structured into 14 modules. Each module, except the last and first modules, has linear residual connections around them. This makes the architecture easier to modify and define. It has the same parameters as the InceptionV3 [23].

## IV. EXPERIMENTAL SETTING

The aforementioned deep learning models are experimented under various experimental settings. The key hyper-parameters have a significant effect on the model’s performance. The fully connected layers of the deep learning models comprises trinity of classification dense layers with 256 input neurons, 128 intermediate neurons and finally 1 output neuron. The first two dense layers of the fully connected layers employ *ReLu* activation function and sigmoid activation function is used at the final output layer with dropout rate of 0.3. Binary cross-entropy loss function guided by *adam* optimizer is entrusted in this deep learning models. Moreover, the Input image size of all the images has been listed in the Table 3.

## V. RESULTS AND DISCUSSION

### A. EVALUATION METRICS

One of the crucial sections of the algorithm is to assess the model for noticing the correctness and execution of various classifiers on the test data and detecting the pre-eminent model from all. True Positive (*TR*_*P*_) or True Negative (*TR*_*N*_) or False Positive (*TR_N_*) or False Negative (*FL_N_*) are used to calculate the accuracy metrics as follows [40].


(1) $$ACC=\frac{TR_{P}+TR_{N}}{TR_{P}+FL_{P}+TR_{N}+FL_{N}}$$

(2) $$Precision=\frac{TR_{P}}{TR_{P}+FL_{P}}$$

(3) $$Recall=\frac{TR_{P}}{TR_{P}+FL_{N}}$$

(4) $$F1-score=\frac{2TR_{P}}{2TR_{P}+FL_{P}+FL_{N}}$$

(5) $$Specificity=\frac{TR_{N}}{TR_{N}+FL_{P}}$$

In this study, Balanced Accuracy (BAC) metrics [41], [42] have been used. BAC is calculated when the dataset is imbalanced and it represents the model accuracy better. It is the average recall obtained in both classes. The precision and specificity are the most important and crucial metrics for medical diagnostics. This is important clinically, as ruling out a diagnosis has a large impact on treatment required or further investigations.

### B. EXPERIMENTAL RESULTS

**FIGURE 5.**
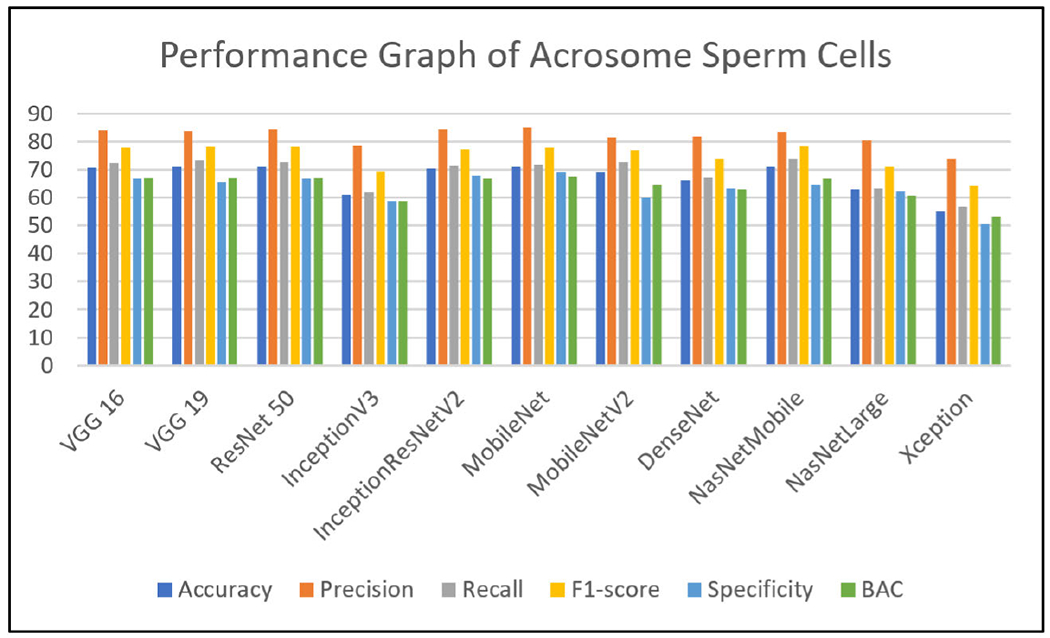
Performance graph of state-of-the-art deep learning models of acrosome sperm cells.

**FIGURE 6.**
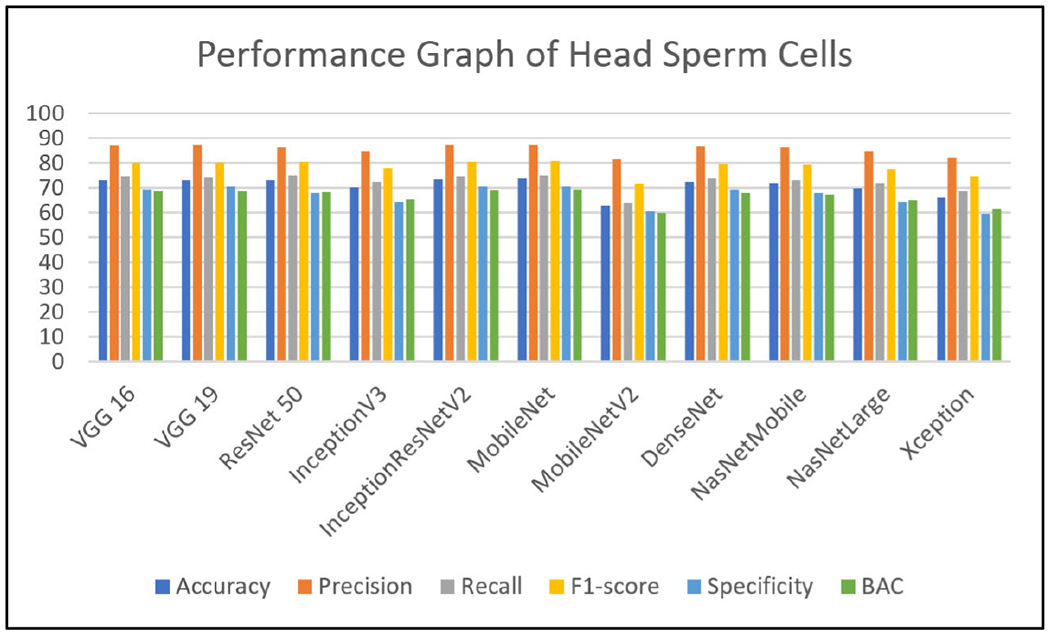
Performance graph of state-of-the-art deep learning models of vacuole sperm cells.

**FIGURE 7.**
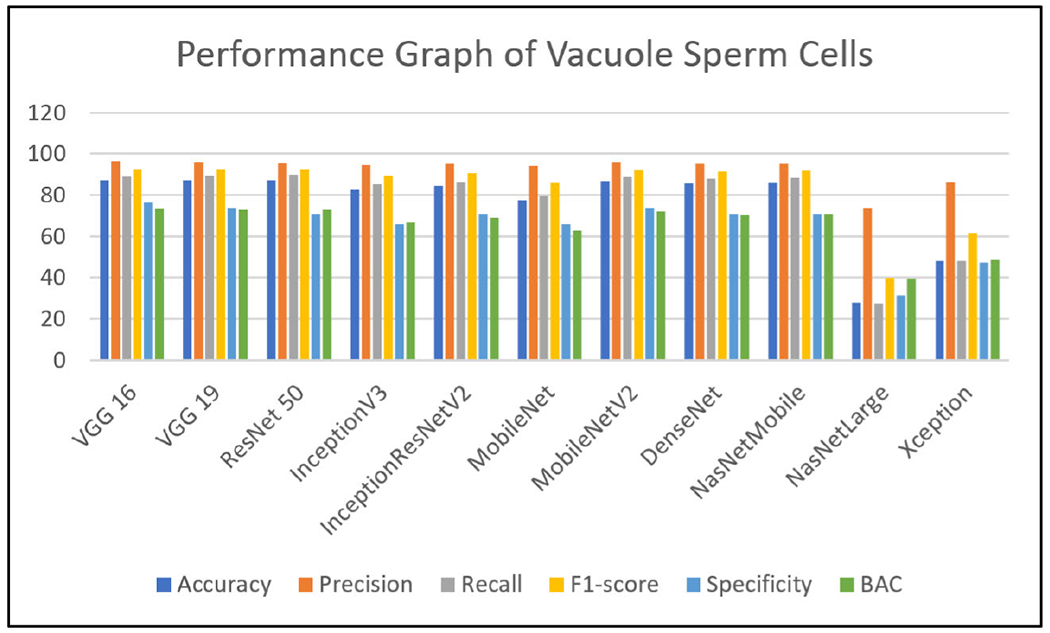
Performance graph of state-of-the-art deep learning models of head sperm cells.

Extensive experiments have been performed using state-of-the-art deep learning models like VGG19 [24], VGG16 [24], ResNet50 [35], InceptionV3 [23], InceptionResNetV2 [25], MobileNet [26], MobileNetV2 [27], DenseNet [28], NASNetMobile [29], NASNetLarge [30] and Xception [31] on all the three labels of the MHSMA dataset [5]. Due to the insufficiency of the training data, various data augmentation techniques (rotation, scaling, and flipping) have been adopted to virtually expand the dataset and intercept overfitting. The evaluating metrics such as accuracy, F1-score, precision, recall and specificity [40] have been assessed to validate and verify the results of the eleven pre-trained architectural models have been discussed in this section. Each model has been trained with a batch size of 34 and epoch size of 25 and model architectures take different periods per epoch for training and testing in particular datasets [5]. It has been observed from the experiments that VGG16 [24], VGG19 [24] and NASNetLarge [30] take maximum average time in the range of 200 to 300 seconds per epoch. InceptionResNetV2 [25], DenseNet [28], ResNet50 [35] and Xception [31] take time in the range of 60 to 80 seconds per epoch. InceptionV3 [23] and NASNetMobile [29] take an average time of 35 seconds per epoch. MobileNet [26] and MobileNetV2 [27] take a minimum time of 18 per epoch. All these deep learning models are processed with an 8 GB RAM and Intel Core i5 1.80 GHz processor in Microsoft Windows 10 operating system environment. The number of false negative, false positive, true negative and true positive of various classifiers has been shown in Table 4. The different metrics have been displayed in Table 5, Table 6 and Table 7. The performance of the various classifiers concerning the accuracy, F1-score, recall, precision, specificity and BAC [41], [42] have been depicted graphically in Figure 5, Figure 6 and Figure 7. Training the very large number of trainable parameters is time intensive and hence, all the convolution layers have been frozen and the models have been trained from scratch.

**FIGURE 8.**
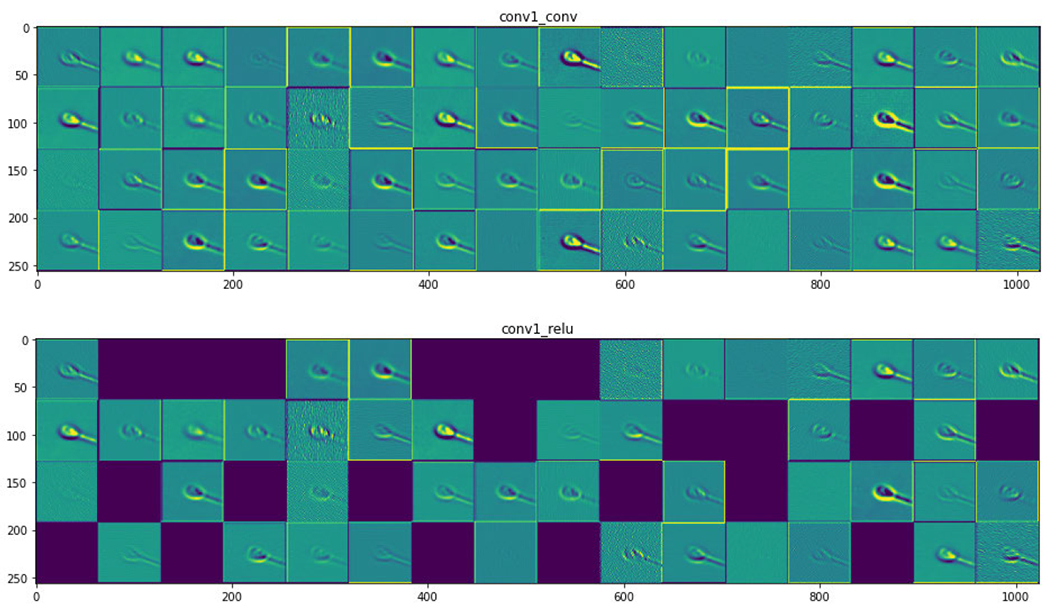
Activation layer for acrosome sperm cells.

**FIGURE 9.**
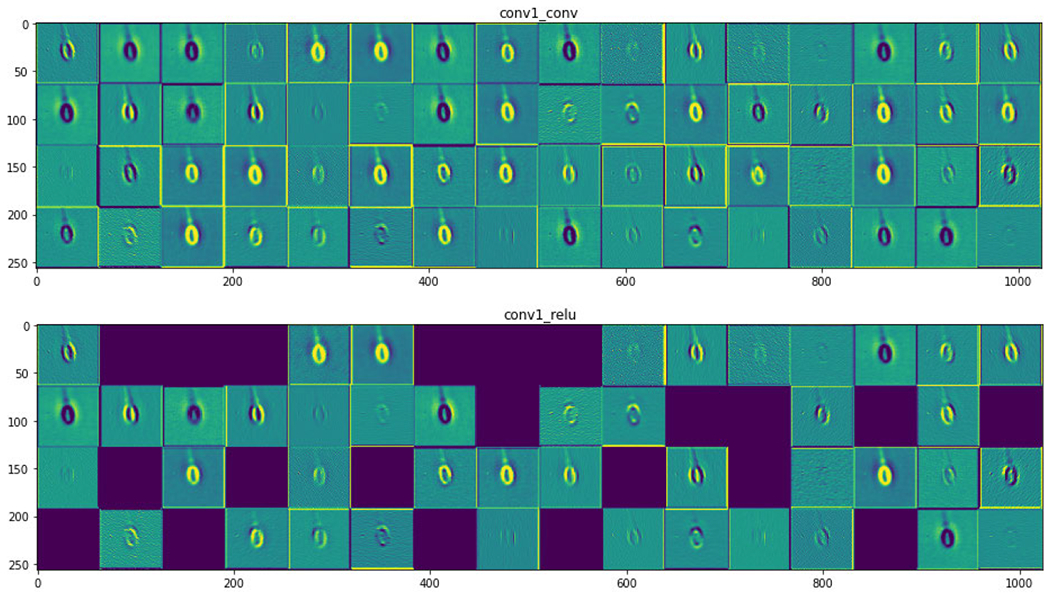
Activation layer for vacuole sperm cells.

**FIGURE 10.**
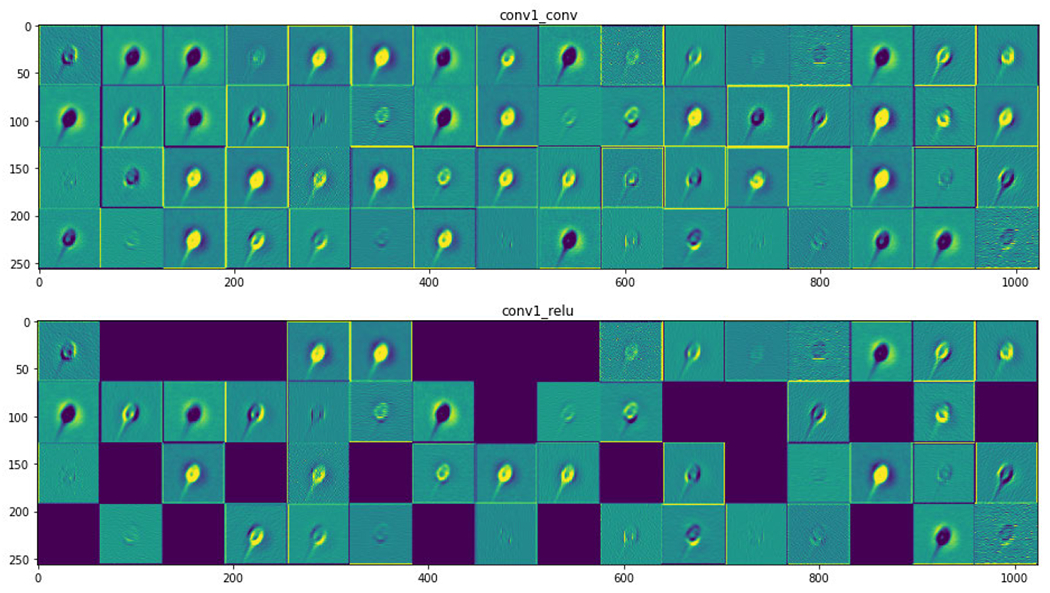
Activation layer for head sperm cells.

Figure 8 shows the neural activation of the layer for acrosome sperm cells. Figure 9 and Figure 10 represent the neural activation of the layer for vacuole and head sperm cells, respectively.

**FIGURE 11.**
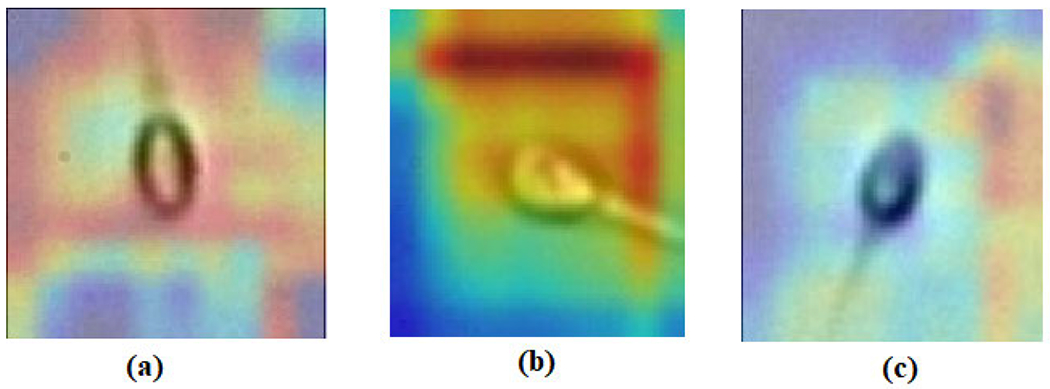
Grad-CAM representation of the sperm cell for (a) Acrosome (b) Vacuole (c) Head.

We have also presented the Gradient-weighted Class Activation Mapping (Grad-CAM) [43] incorporating the gradients of attack concept, flowing into the final convolution layer for producing a rough localization map featuring principal regions in the image for forecasting the concept. Figure 11 represents the Grad-CAM visualization of the sperm cells for abnormality check of acrosome, head and vacuole part of the cell.

The experimental results presented in Table 5 are prevalent to the acrosome label for finding infertility due to the malformation of the acrosome part of the sperm cell. The results show that VGG19 [24] and ResNet50 [35] models performed well with an accuracy of 71%, and BAC 66.94% and 67.12%, respectively. Whereas, MobileNet [26] and NASNetMobile [29] have secured 71% accuracy with the precision value 85% and 83.51%, respectively. However, Xception [31] and NASNetLarge [30] have reported relatively low accuracy of 55% and 63% compared with other state-of-the art models.

Any significant deviation in terms of performance of all models has been observed while accuracy is taken into consideration. With a mean accuracy of 67%, it is interesting to notice that these models independently do not perform well when detecting the acrosome. This can be attributed to the fact that the acrosome is situated at the tip of the head and has a much smaller area to accurately detect for CNNs. It may be noted that the resolution of the sperm images does not allow for accurate detection of a region as small as the acrosome by any of the CNNs. One remedy to this problem could be to use these state-of-the-art CNNs without pre-training by ImageNet weights. Another solution could be to use a bagging approach and using the ensemble power of all CNNs to detect the acrosome in a majority voting scenario.

The mean value of precision attained overall models in Table 5 is 81.92%. This is a promising metric than accuracy as the models can detect correctly the presence of acrosome. It has been found that VGG16 [24], ResNet50 [35], InceptionResNetV2 [25] and MobileNet [26] achieved precision more than 84%. This could be due to the reason that VGGs have a lot of parameters to be trained which come in handy for correct classification of the acrosome, and that residual skip-connections in the case of ResNet50 [35] and InceptionResNetV2 [25] allow for the preservation of features to be learned by different layers to achieve better positive classifications. In the case of MobileNet [26], since it incorporates depth-wise separable convolutional layers, the number of channels affects its performance. Normally, with color images, there are three channels (red, green and blue), however, in our application, we only have a single channel (gray-scale images). Due to the single-channel along with the depth-wise separable convolution, MobileNet [26] may outperform other network models relying on convolutional layers. The performance of MobileNet [26] is not seen to peak (due to the reduced number of parameters), but also it does not stoop so low to be the worst performing model in Table 7. Moreover, Xception [31] also makes use of depth-wise separable convolutions and it also employs regularizes such as dropout and L2 regularization or weight decay as reported and hampers its performance.

In terms of recall (sensitivity) values reported in Table 5, it has been observed that the average recall value over all models is 68.84% and hence, the models are not efficient to detect acrosome. This is owing to the fact that the reported accuracy scores of models on the area covered by the acrosome. Even in the existence of acrosome, it is barely visible in such low dimensions and the models are barely able to keep up with all the samples that have the acrosome. The maximum values of recall only go as high as 73.71%. The models are seen to struggle to detect an absence of the acrosome due to the small low dimensional regions in the samples of the dataset. Xception [31] seems to struggle the most by being able to correctly predict only 50% of all the negatives, and the reasons due to the regularizations.

Table 6 shows the results of models trained on the vacuole label for finding infertility due to the malformation of the vacuole part of the sperm cell. It has been observed that VGG16 [24], VGG19 [24] and ResNet50 [35] models have performed with an accuracy of 87.33%, whereas BAC score is 3.14%, 72.95% and 72.76% for VGG16 [24], VGG19 [24] and ResNet50 [35], respectively. These values altogether look more promising than those found in Table 5. This is due to the region of detection being bigger when we consider the vacuole. The vacuole is the concavity that extends from the surface of the sperm head to the nucleus through the acrosome. This is distinctive (the intermediate neural activations are as shown in Figure 8, 9 and 10) and a higher mean value of almost all the metrics is found when compared with Table 4. It seems that the models NASNetLarge [30] and Xception [31] are unable to detect the features of the vacuole from the dataset and hence reduce their values of all metrics. MobileNet [26] may have the fastest procedure but it comes with the tradeoff of accuracy and other metrics in a majority of the scenarios. We find a similar trend of better accuracy for ResNet50 [35] and the VGGs [24], the reason being the skip-connections and higher number of parameters, respectively. In this regard, InceptionResNetV2 [25] does not disappoint either due to its skip-connections.

The mean precision values obtained using the deep models are found to be 92.5%. However, this could be very deceptive too due to Xception [31] and NASNetLarge [30] achieved very low accuracy. This is interesting to note that VGG16 [24] is reported with promising accuracy of as high as 96.2%.

In terms of recall, the precision values for Xception [31] and NASNetLarge [30] are deceptive, as they reported low recall values of 48% and 27%, respectively. The rest of the models attain satisfactory recall value with an average of 87%. The high values of the metrics of precision and recall lead to high values of the F1-score metric as well. However, the metric values drop slightly when we consider the specificity. The mean value of specificity (except Xception [31] and NASNetLarge [30]) is found to be 71%. Due to the lower resolution of the imaging of the sperms, the detection of vacuoles is an uphill task and often the edges may look like vacuoles. It leads to miss-classifications in an absence of a vacuole which is evident from the experimental results.

Table 7 shows the results of models trained on the head label for finding infertility due to the malformation of the head part of the sperm cell. It may be noted that VGG19 [24] and ResNet50 [35] model outperformed the rest of the models with an accuracy of 73.34% and 73.36%, respectively. The BAC score is obtained as of 68.8% and 69.06% for VGG19 [24] and ResNet50 [35], respectively and also the precision values are 87.16% for VGG19 [24] and 87.23% for ResNet50 [35].

We have been basing our arguments on the better and worse performance of the models overall metrics due to the lower resolution of the images. It is worth noting that the head of the other classes of vacuole and acrosome, being the largest in terms of area covered in the image, are relatively easier to detect by the models. However, the metric values reported in Table 7 are relatively lower than in Table 6. This is because the structure of the head does not curve back in too much before we reach the tail. This often means that the head is deformed and does not have as much volume as it should normally have and this distinction is very subtle for models to accurately pick up. There are not enough instances in the dataset that help the models to learn just how many angles between the tail and the start of the head are required for it to be deemed a normal head, or vice-versa for deeming it an abnormal head. Nevertheless, the average accuracy of all the models for the detection of a normal head is 70.75%. It can be said from the experimental outcome that the state art deep learning models correctly predict the abnormality of the head in a sperm approximately 3 times out of 4.

Precision values of all models look promising as the mean is 85.38% and there is not much distinction between the values of precision attained by each model as the range lies between 81.29% and 87.23%. The reason for high precision values is that it is very distinctive and it becomes easier for the models to predict the positive class.

The average value for the recall is relatively lower than expected as of 72.27% due to the subtle differences between the abnormality of the head and its normality. Similar to precision, the range of values of recall is very low and hence there are no such models that perform distinctively better or worse to analyze. It may be noted that F1-score being the harmonic mean of precision and recall, are justified solely through mathematical means and the reasoning of the values attained for precision and recall individually.

The average value of specificity is reported low as 66.67% owing to the subtle variation in the case of abnormal heads (related to the slight angular differences between the head and the tail at the contact point), it becomes a paramount task to correctly predict whether a sperm has an abnormal or normal head. In this study, no such models found were to perform well as the highest specificity attained is 70.37% by VGG19 [24] and ResNet50 [35].

Overall, the performance of VGG19 [24] outperforms VGG16 [24] as VGG19 [24] has extra trinity of layers *i.e.*, 3 × 3 Conv 256, 3 × 3 Conv 512 and 3 × 3 Conv 512 layers and therefore trained well with VGG19 [24]. ResNet50 [35] architecture has also performed well as ResNet architecture has skip connections which allows gradients to easily flow across the layer and even the bottom-most layer receives activations from the top layer which assist in training very deep networks. Similarly, InceptionResNetV2 [25] has outperformed InceptionV3 [23] as it is deeper than the InceptionV3 [23] model and also Inception blocks have been simplified containing fewer parallel towers than InceptionV3 [23]. Therefore it is more powerful and accurate. NASNetLarge [30] under-performed in terms of accuracy as compared to all the other algorithms used in the investigation as NASNetLarge [30] does not consist of the linear sequence of modules. MobileNet [26] performed better with the acrosome and head part of the cell while MobileNetV2 [27] achieved higher accuracy than MobileNet [26] with the vacuole part of the sperm.

Moreover, the statistical *t*-tests [44] have been performed for all the metrics as shown in Table 5–7 using the following.

(6) $$t=\frac{\bar{X_{1}}-\bar{X_{2}}}{\sqrt{\left(\frac{{\left(N_{1}-1\right)}^{2}S_{1}+{\left(N_{2}-1\right)}^{2}S_{2}}{N_{1}+N_{2}-1}\right)\left(\frac{1}{N_{1}}+\frac{1}{N_{2}}\right)}}$$

Here, *X*_1_ and *X*_2_ are the means of the accuracy of the acrosome and the vacuole, respectively, for all the deep learning models with variance *S*_1_ and *S*_2_ in each set. *N*_1_ and *N*_2_ are the number of samples in each set. The *p*-values of the *t*-tests are computed as shown in Table 8 and it signifies statistical significance of the model if the *p*-value is less than 0.05.

## VI. CONCLUSION

In this manuscript, the sperm cells have been analyzed for predicting their fertility. The paper’s finding promotes an understanding of the normality or abnormality of the cells due to the normal or abnormal acrosome, vacuole, or head part of the cell in a real-time and rapid way. The study defeats the traditional testing methods of the sperm cells, which is very time-consuming. The recognized patterns can be very effective in the medical field for determining the fertility of the sperm cells. Different deep learning techniques have been employed for assessing the normality of the sperm cells. It has been observed from the experimental results that both VGG19 and ResNet50 models achieved an accuracy of 71%, 87.33%, 73% for acrosome, vacuole and head label, respectively. The results in this current research show an impact on the different health organizations and the research community. However, despite performing better, the suggested techniques cannot attain ideal F1-score and accuracy on every label of the MHSMA sperm dataset. Moreover, the size of the current dataset is one of the enormous existing available datasets, but it needs to be enlarged for further experimental assessment of the algorithms. An ensemble of deep learning architectures may be a potential solution to exploit relatively large datasets. However, ensemble-based deep learning models often suffer from convergence problems and their complexity increases with the increase in the number of deep learning architectures in the ensemble models. It remains to investigate with a limited number of deep learning architectures in the ensemble model. Authors are currently engaged in this direction of research.

## Figures and Tables

**TABLE 1.** Dataset description.

Set	Class	Positive	Negative
Training Set	Vacuole	830	170
	Head	727	273
	Acrosome	699	301
	Tail and Neck	954	46
Validation Set	Vacuole	209	31
	Head	176	64
	Acrosome	174	66
	Tail and Neck	233	7
Test Set	Vacuole	262	38
	Head	219	81
	Acrosome	213	87
	Tail and Neck	284	16

**TABLE 2.** Dataset split details [5].

	Number of Images	Split Percentage
Training Set	1000	69.94%
Validation Set	240	15.58%
Test Set	300	19.48%

**TABLE 3.** Size of the input tensors.

Models	Input Tensor size
VGG16	224 × 224 × 3
VGG19	224 × 224 × 3
ResNet50	224 × 224 × 3
InceptionV3	299 × 299 × 3
InceptionResNetV2	299 × 299 × 3
MobileNet	224 × 224 × 3
MobileNetV2	224 × 224 × 3
DenseNet	224 × 224 × 3
NasNetMobile	331 × 331 × 3
NasNetLarge	331 × 331 × 3
Xception	299 × 299 × 3

**TABLE 4.** Performance of the state-of-the-art deep learning models using a confusion matrix.

Model	Acrosome				Vacuole				Head			
	
	*TR_P_*	*TR_N_*	*FL_P_*	*FL_N_*	*TR_P_*	*TR_N_*	*FL_P_*	*FL_N_*	*TR_P_*	*TR_N_*	*FL_P_*	*FL_N_*
VGG16 [24]	154	58	29	59	233	29	9	29	163	56	25	56
VGG19 [24]	156	57	30	57	234	28	10	28	163	57	24	56
ResNet50 [35]	155	58	29	58	235	27	11	27	164	57	24	55
InceptionV3 [23]	132	51	36	81	223	25	13	39	158	52	29	61
InceptionResNetV2 [25]	152	59	28	61	226	27	11	36	162	57	24	57
MobileNet [26]	153	60	27	60	208	25	13	54	164	55	26	55
MobileNetV2 [27]	155	52	35	58	232	28	10	30	139	49	32	80
DenseNet [28]	143	55	32	70	230	27	11	32	161	56	25	58
NasNetMobile [29]	157	56	31	56	231	27	11	31	160	55	26	59
NasNetLarge [30]	135	54	33	78	72	12	26	190	157	52	29	62
Xception [31]	121	44	43	92	126	18	20	136	150	48	33	69

**TABLE 5.** Performance of the state-of-the-art deep learning models for acrosome sperm cells.

Model	Accuracy	Precision	Recall	F1-score	Specificity	BAC
VGG16 [24]	70.67	84.15	72.30	77.78	66.67	66.86
VGG19 [24]	71.00	83.87	73.24	78.20	65.52	66.94
ResNet50 [35]	71.00	84.24	72.77	78.09	66.67	67.12
InceptionV3 [23]	61.00	78.57	61.97	69.29	58.62	58.60
InceptionResNetV2 [25]	70.33	84.44	71.36	77.35	67.82	66.81
MobileNet [26]	71.00	85.00	71.83	77.86	68.97	67.50
MobileNetV2 [27]	69.00	81.58	72.77	76.92	59.77	64.43
DenseNet [28]	66.00	81.71	67.14	73.71	63.22	62.86
NASNetMobile [29]	71.00	83.51	73.71	78.30	64.37	66.76
NASNetLarge [30]	63.00	80.36	63.38	70.87	62.07	60.63
Xception [31]	55.00	73.78	56.81	64.19	50.57	53.07

**TABLE 6.** Performance of the state-of-the-art deep learning models for vacuole sperm cells.

Model	Accuracy	Precision	Recall	F1-score	Specificity	BAC
VGG16 [24]	87.33	96.28	88.93	92.46	76.32	73.14
VGG19 [24]	87.33	95.90	89.31	92.49	73.68	72.95
ResNet50 [35]	87.33	95.53	89.69	92.52	71.05	72.76
InceptionV3 [23]	82.67	94.49	85.11	89.56	65.79	66.78
InceptionResNetV2 [25]	84.33	95.36	86.26	90.58	71.05	69.11
MobileNet [26]	77.67	94.12	79.39	86.13	65.79	62.88
MobileNetV2 [27]	86.67	95.87	88.55	92.06	73.68	72.07
DenseNet [28]	85.67	95.44	87.79	91.45	71.05	70.60
NASNetMobile [29]	86.00	95.45	88.17	91.67	71.0	71.00
NASNetLarge [30]	28.00	73.47	27.48	40.00	31.58	39.70
Xception [31]	48.00	86.30	48.09	61.76	47.37	48.99

**TABLE 7.** Performance of the state-of-the-art deep learning models for the head sperm Cells.

Model	Accuracy	Precision	Recall	F1-score	Specificity	BAC
VGG16 [24]	73.00	86.70	74.43	80.10	69.14	68.35
VGG19 [24]	73.33	87.17	74.43	80.30	70.37	68.80
ResNet50 [35]	73.67	87.23	74.89	80.59	70.37	69.06
InceptionV3 [23]	70.00	84.49	72.15	77.83	64.20	65.25
InceptionResNetV2 [25]	73.00	87.10	73.97	80.00	70.37	68.55
MobileNet [26]	73.00	86.32	74.89	80.20	67.90	68.16
MobileNetV2 [27]	62.67	81.29	63.47	71.28	60.49	59.64
DenseNet [28]	72.33	86.56	73.52	79.51	69.14	67.84
NASNetMobile [29]	71.67	86.02	73.06	79.01	67.90	67.13
NASNetLarge [30]	69.67	84.41	71.69	77.53	64.20	65.01
Xception [31]	66.00	81.97	68.49	74.63	59.26	61.50

**TABLE 8.** p-value for all the metrics.

	Accuracy	Precision	Recall	F1-score	Specificity	BAC
Acrosome and vacuole	0.074	0.000089	0.084	0.054	0.3121	0.3244
Vacuole and head	0.1785	0.00185	0.184	0.155	0.376798	0.404
Acrosome and head	0.039	0.00445	0.5208	0.249	0.0455	0.076

## Data Availability

The dataset can be downloaded with following link: https://github.com/soroushj/mhsma-dataset/archive/refs/heads/master.zip.
